# Rectal Sedation With Ketamine and Midazolam in the Management of Uncooperative Children During Dental Treatment: A Case Series and Method Description

**DOI:** 10.7759/cureus.54825

**Published:** 2024-02-24

**Authors:** Hasan Alzoubi, Samar Kabbani, Ahmad Taleb, Nada Bshara, Mohamed K Altinawi, Mohammed Bashier Almonakel, Saleh Al Kurdi

**Affiliations:** 1 Department of Pediatric Dentistry, Damascus University, Damascus, SYR; 2 Department of Anesthesia and Reanimation, Damascus University, Damascus, SYR

**Keywords:** rectal sedation, ketamine, midazolam, uncooperative children, dental treatment

## Abstract

Background

In pediatric dentistry, sedation aims to eliminate anxiety to facilitate the completion of dental procedures. Sedation in children is a multidimensional field that includes the child, parents/guardians, and the health care team. The rectal route is generally painless, making it suitable for children who are afraid of needles. This route has several advantages over the oral route, including reduced patient cooperation requirements, a faster and more predictable onset, and less physical trauma than the intravenous and intramuscular routes. This case series aimed to evaluate the effectiveness and success rate of rectal sedation with ketamine and midazolam in the management of uncooperative children during dental treatment.

Case presentation

Ten healthy children with definitely negative behavior were enrolled in this study. Each child was given 7 mg/kg of ketamine in combination with midazolam 0.1 mg/kg by the rectal route. The mean onset sedation time was 9.5 minutes, and pulpotomy procedures were done. Behavioral response was monitored throughout treatment using the Ohio State University Behavioral Rating Scale (OSUBRS), and the depth of sedation was measured using the University of Michigan Sedation Scale (UMSS). The Houpt General Behavior Scale was used to estimate the treatment success rate based on the overall behavior rating. All 10 cases showed good anxiolysis and cooperation following rectal administration, with no side effects observed.

Conclusions

Rectal administration of ketamine in combination with midazolam may be considered a reliable method in the management of uncooperative children during dental treatment. No adverse effects were observed during or after the sedation procedure.

## Introduction

Due to dental fear and anxiety, children need a larger number of sessions and a longer time to complete their dental treatment, and they also show poor cooperation during dental treatment, which negatively affects the results of treatment and creates professional pressure on the dental staff [1]. There is a wide range of behavior management techniques, from basic non-pharmacological techniques to advanced non-pharmacological techniques, as well as pharmacological techniques including general anesthesia and sedation [2].

The term "pharmacological sedation" refers to a method where a patient's central nervous system is suppressed by one or more drugs, thereby decreasing his awareness of his surroundings. Based on the extent of central nervous system depression, the American Association of Pediatric Dentistry (AAPD) has classified sedation levels into three categories: minimal, moderate (conscious), and deep [3].

Numerous sedative routes, including oral, nasal, rectal, intravenous, and intramuscular ones, have been suggested for the treatment of children with negative behavior in dental clinics [4]. Opening an intravenous line in young children is painful, time-consuming, and requires special skills. For these reasons, the possibility of inducing sedation by routes other than intravenously is more important, especially if the procedure is repeated [5,6]. Children and their parents often find rectal administration less threatening than the nasal, intramuscular, or intravenous route, and rectal medications have been used for sedation for many years and are particularly beneficial in pediatric patients [7].

When a drug is administered rectally, it usually has a higher bioavailability, faster effect, and shorter peak than the oral route. Rectal drug administration also tends to reduce nausea and prevents any loss of the medication due to vomiting compared to the oral route [8]. Furthermore, the rectal routes undergo two-thirds of the primary metabolism because the venous drainage of the rectum is two-thirds systemic (middle and inferior rectal vein) and one-third portal (superior rectal vein) [9]. This means that the drug will reach the circulatory system at a higher concentration and with less change.

Midazolam is a relatively short-acting benzodiazepine that is anxiolytic, hypnotic, and anticonvulsant. Sedation using midazolam offers a method that may be safe, but it is often ineffective, as midazolam alone is not sufficient to complete various therapeutic or diagnostic procedures [10]. Ketamine provides sedation, analgesia, and memory loss. Ketamine can be administered through various routes, including intravenous, intramuscular, intranasal, oral, or rectal injection, and is considered safe and effective [11].

With this background, the effectiveness and safety of rectal administration of ketamine in combination with midazolam was evaluated in the management of uncooperative children during dental treatment.

## Materials and methods

The Ethics Committee of Damascus University, Syria's Scientific Research and Postgraduate Board, gave its approval to this study (IRB No. UDDS2805-22112023/SRC-1550). Ten healthy children aged between three and five years old presented to the Department of Pediatric Dentistry, Damascus University. All parents and guardians provided written informed consent regarding the procedures that will be carried out. All children were definitely negative (Rating 1: refusal of treatment, crying forcefully, fearful, or any other overt evidence of extreme negativism) according to Frankl's behavioral scale [12]. The difficulty of modifying these behaviors was evaluated by Master's students in the Department of Pediatric Dentistry using simple non-pharmacological behavioral management methods or advanced pharmacological behavioral management methods such as nitrous oxide sedation and oral sedation methods.

**Figure 1:**
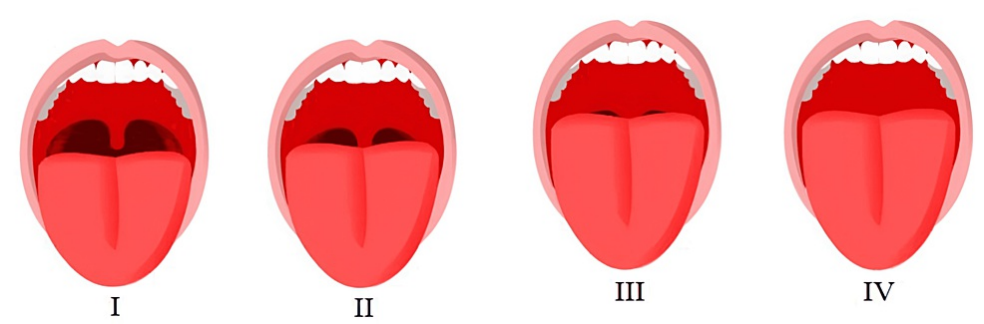
Mallampati classification Source [14]: under the terms of the Creative Commons Attribution-NonCommercial-NoDerivatives 4.0 International (CC BY-NC-ND 4.0) ( http://creativecommons.org/licenses/by-nc-nd/4.0/).

All children were healthy and were classified (American Anesthesia Association (ASA) I: a normal healthy patient; example: Fit, nonobese) according to the ASA classification [13], and classified (I: fully visualize the faucial pillars, soft palate, and uvula; or II: full visualization of the faucial pillars and soft palate, unable to visualize the uvula, as it is obscured by the base of the tongue) according to the Mallampati classification for evaluating the airways (Figure 1) [14]. Any child who had digestive system disorders, high fever, enlarged tonsils, influenza symptoms, snoring, or mouth breathing was excluded.

It was confirmed that there is no contraindication for the use of any sedative medications or local anesthetics (midazolam, ketamine, or lidocaine used in local anesthesia) based on the medical history by asking the child’s parents/guardian. All children underwent a general medical examination by the pediatrician, who, if there is no objection to the child’s sedation, will provide them with a written consultation confirming that the patient is a candidate to receive sedation and that there is no objection to the child receiving sedation medications.

After conducting a health assessment of the child by the anesthesiologist at the Oral and Maxillofacial Surgery Hospital at the Faculty of Dentistry at Damascus University, and examining the airway and chest by inspection and auscultation, informed written consent was obtained from the parents/guardian on the procedures that will be taken and for their child’s participation in the study after providing a brief explanation of the procedures to be performed and the benefits expected.

Sedation instructions were given to the parents/guardian prior to the sedation session, along with fasting instructions (not eating or drinking anything for six hours before the appointment time, including soup, sweets, milk, tea, coffee, or chewing gum; it is important that drink plenty of water up until two hours before appointment time).

**Figure 2:**
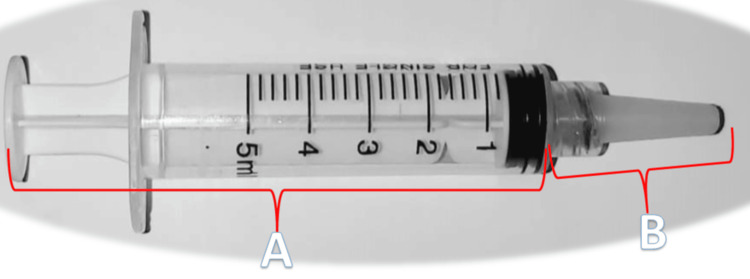
The rectal injection after mounting the drilled tube (A): Intramuscular injection syringe; (B): The novel and drilled tube

The child's weight was measured and the child's basic vital signs (pulse rate, oxygen saturation (SpO2), systolic blood pressure, and diastolic blood pressure) were recorded before drug administration. The sedative drugs ketamine 7 mg/kg (Ketamine Rotexmedica 50 mg/ml, Arzneimittelwerk GmbH Rotexmedica, Germany) in combination with midazolam 0.1 mg/kg (Midazolam 5 mg/ml, AVENZOR, Damascus, Syria) was administered rectally (as the child lay on his back with the buttocks tightly pressed for a minute after the injection to avoid loss of the drug) via a specially designed, novel, and drilled tube (Figure 2) (the amount of entry is 3-4 cm after applying 2% lidocaine gel as a lubricant and anesthetic).

The children were monitored according to the basic guidelines of the American Academy of Pediatric Dentistry (AAPD), as the oxygen saturation, systolic pressure (SB), diastolic pressure (DB), and pulse rate were recorded every five minutes until the end of treatment. When symptoms of sedation began to appear (nystagmus), local anesthesia and rubber dam isolation were applied, and dental treatment was started (while the child was still under the influence of the sedative), taking into account the placement of a special headrest from the beginning of treatment to support the neck to keep the airways open.

Behavioral response was monitored throughout treatment using the Ohio State University Behavioral Rating Scale (OSUBRS) (score 1: calm, score 2: crying without resistance, score 3: movement with resistance, score 4: movement with resistance and crying). The depth of sedation was measured using the University of Michigan Sedation Scale (UMSS) (score 0: awake, score 1: slightly sedated, responds to or voice prompts, score 2: moderately sedated, responds to tactile stimulations or verbal commands, score 3: deeply sedated, responds only to strong physical stimulations, score 4: unresponsive).

The Houpt General Behavior Scale was used to estimate the treatment success rate based on the overall behavior rating. The scale has six scores, ranging from 1 (fail) to 6 (excellent), with scores 1 and 2 indicating a failure of the sedation process and the remaining scores indicating a success (Table 1).

**Table 1:** Houpt general behavior scale Source: [15] (This is an open-access article distributed under the Creative Commons Attribution License, which permits unrestricted use, distribution, and reproduction in any medium, provided the original work is properly cited.)

Score	Type of behavior	Behavioral assessment	Result of the sedation system
1	Failure	It was not possible to do the treatment at all.	Failure
2	Bad	Partial treatment was done.	
3	Moderate	completed treatment but intermittent	Success
4	Good	Moderate crying or movement.	
5	Very good	Some crying and limited movement.	
6	Excellent	No movement or crying.	

Following treatment, the child was placed in a comfortable area until he satisfied the requirements for recovery from sedation: the patient met score 1 or 2 on the modified Vancouver scale (Table 2), and vital signs were within normal ranges. On the evening of the treatment day, the child's parents were also contacted in case any side effects occurred.

**Table 2:** Modified Vancouver scale Adapted from: [16]

Score	Behavioral assessment
1	Completely awake.
2	The patient responds to verbal questions (eyes open).
3	The patient does not respond to verbal questions (eyes open).
4	The patient does not respond to verbal questions (eyes closed).
5	The patient is aroused by slight agitation (eyes closed).
6	The patient is not aroused by slight agitation (eyes closed).

## Results

This study included 10 healthy children (70% females and 30% males) aged between three and five years old (mean 3.8±0.8) The mean onset sedation time was (9.5±1.3 minutes), treatment time (20.25±2.43 minutes), and recovery time (12.30±3.30 minutes) (Table 3). No complications were recorded following sedation. Regarding vital signs, the mean oxygen saturation was (98.3, 97.4, 98.1), pulse rate (96.00, 94.67, 95.47), SB (10.52, 11.10, 10.83), and DB (6.4, 6.5, 6.53) before, during and after treatment, respectively (Table 4).

**Table 3:** The mean ages and onset of sedation, treatment, and recovery times

Variables	Mean	SD
Ages	3.8 (years)	0.8
Onset sedation time	9.5 (minutes)	1.3
Treatment time	20.25 (minutes)	2.43
Recovery time	12.30	3.30

**Table 4:** The mean vital signs before, during, and after treatment PR: pulse rate; SBP: systolic blood pressure; DBP: diastolic blood pressure

Variables	Stage	Mean	SD
SpO2	Before treatment	98.3	1.04
	During treatment	97.4	1.25
	After treatment	98.1	1.07
PR	Before treatment	96.00	2.55
	During treatment	94.67	3.89
	After treatment	95.47	2.14
SBP	Before treatment	10.52	0.23
	During treatment	11.10	0.27
	After treatment	10.83	0.28
DBP	Before treatment	6.4	0.26
	During treatment	6.5	0.25
	After treatment	6.53	0.22

The child's level of behavioral response was continuously monitored during treatment using the Ohio State University Behavioral Rating Scale (OSUBRS), as all children (100%) showed a score of 1 (calm without movement) according to this scale. As for the depth of sedation, it was evaluated according to the University of Michigan scale (UMSS; 7 children (70%)) showed a score of 3 (deep sedation, deep asleep, responding only to strong physical stimuli) while three children (30%) showed a score of 2 (moderate sedation, sleepy, responsive to light or tactile stimuli or verbal orders) (Table 5).

**Table 5:** The results of the OSUBRS, UMSS, and Houpt scales OSUBRS: Ohio State University Behavioral Rating Scale; UMSS: University of Michigan Sedation Scale

Scores	Scales		
	OSUBRS	UMSS	Houpt
Score 0	-	0 (0%)	-
Score 1	10 (100%)	0 (0%)	0 (0%)
Score 2	0 (0%)	3 (30%)	0 (0%)
Score 3	0 (0%)	7 (70%)	0 (0%)
Score 4	0 (0%)	0 (0%)	0 (0%)
Score 5	-	-	2 (20%)
Score 6	-	-	8 (80%)

At the end of the treatment, the success rate of the treatment was evaluated using the Houpt general behavior scale. The success rate was 100%, and two children (20%) showed some crying and limited movement (score 5) while eight children (80%) did not show any crying or movement (score 6) (Table 5).

## Discussion

Young pediatric patients who need dental treatments suffer from high levels of fear and anxiety; as Son et al. reported the prevalence of dental fear was 34.85% [17]. The Sarapultseva et al. study found that 93.8% of Russian children visiting dental clinics mostly suffer from moderate levels of dental fear and anxiety [18], and the Dahal et al. study also found that the prevalence of severe dental fear was 44.7%, moderate dental fear was 28.8%, while the mild dental fear was 26.5% [19].

In general, treatment under general anesthesia is necessary for these patients, but when dental treatment involves a small number of units and a short operating time, alternative methods, such as conscious sedation with different routes (oral, intranasal, and rectal), have been introduced [20]. Giving the drug rectally is beneficial for patients who are unable to swallow, and rectal methods are a faster and safer alternative or alternative to intravenous or subcutaneous injections in various cases [21].

There are several reasons why rectal administration is preferable to the oral route [8]: suitable for administering medications to patients with swallowing problems or decreased consciousness, rectal absorption is not affected by vomiting or gastrointestinal disease, and is a good option when the sensory properties of the oral dose (taste, color, smell) are undesirable. Drugs absorbed from the rectum bypass the liver and go directly into the systemic circulation, which increases the bioavailability of the drug compared to the oral route.

No differences in the efficacy of drugs were observed when administered orally and rectally, resulting in the rectal route being more beneficial [22]. In fact, in the studied cases, blood oxygen saturation did not drop below 95%, and no side effects appeared during and after sedation.

Regarding the used sedative drugs, midazolam is a sedative-hypnotics drug that causes sedation or hypnosis depending on the dose of the drug given and the patient’s response to it (low doses of these drugs produce a sedative effect that causes a degree of drowsiness and ataxia while high doses lead to It leads to hypnosis) [23,24], while ketamine causes a state of conscious sedation rather than central nervous system depression [25]. Ketamine provides excellent amnesia and analgesia and maintains muscle coordination, airway reflexes, and spontaneous breathing with its distinct analgesic effect in adults and children [26]. Ketamine also has anti-inflammatory effects and antidepressant activity [27].

According to this study, the combination of midazolam and ketamine made dental treatment safer and more reliable for all pediatric patients, and none of the cases showed treatment interruption due to excessive movement or resistance. This can be attributed to enhancing the sedative effect and benefiting from the properties of all the medications used, meaning that they work to improve the desired effects and eliminate the side effects.

In this study, ketamine allowed a treatment time of 30 minutes when the recovery time was taken into account, and this was in accordance with the Yoshino et al. study, which found that 5 mg/kg ketamine, in combination with midazolam 0.1 mg/kg, gave a treatment time of 30 minutes while obtaining good cooperation and eliminating anxiety in children and was considered a safe and reliable method [28].

In this study, the combination of ketamine with midazolam showed 100% success as all dental treatments were completed and this is in accordance with the Grossmann et al. study, which found that combining ketamine 6 mg/kg with midazolam 0.5 mg/kg during pediatric burn dressing procedures for approximately 30 minutes provides optimal conditions in terms of pain relief, efficacy, recovery, and patient safety [29].

This study describes a case series of the effectiveness of rectal ketamine during dental treatment. This is the most important limitation of the current study, as randomized controlled clinical studies must be conducted to evaluate the effectiveness of administering ketamine by this route compared to the oral, intramuscular, or intravenous route.

## Conclusions

The combination of ketamine with midazolam rectally may be considered an effective regimen in the management of uncooperative children during dental treatment. However, more clinical studies with larger sample sizes and evaluating the acceptability of giving sedative drugs in this way to both the parents and the child are still required.
